# Advances in Hydrogel-Based Microfluidic Blood–Brain-Barrier Models in Oncology Research

**DOI:** 10.3390/pharmaceutics14050993

**Published:** 2022-05-05

**Authors:** Ankur Sood, Anuj Kumar, Atul Dev, Vijai Kumar Gupta, Sung Soo Han

**Affiliations:** 1 School of Chemical Engineering, Yeungnam University, 280 Daehak-ro, Gyeongsan 38541, Korea; ankursood02@gmail.com; 2 Institute of Cell Culture, Yeungnam University, 280 Daehak-ro, Gyeongsan 38541, Korea; 3 Division of Gastroenterology & Hepatology, School of Medicine, Institute for Regenerative Cures, University of California Davis, 2921 Stockton Boulevard, Sacramento, CA 95817, USA; atulinst@gmail.com; 4 Biorefining and Advanced Materials Research Center, Scotland’s Rural College, Edinburgh EH9 3JG, UK; vijai.gupta@sruc.ac.uk

**Keywords:** blood–brain barrier, microfluidics, hydrogels, oncology

## Abstract

The intrinsic architecture and complexity of the brain restricts the capacity of therapeutic molecules to reach their potential targets, thereby limiting therapeutic possibilities concerning neurological ailments and brain malignancy. As conventional models fail to recapitulate the complexity of the brain, progress in the field of microfluidics has facilitated the development of advanced in vitro platforms that could imitate the in vivo microenvironments and pathological features of the blood–brain barrier (BBB). It is highly desirous that developed in vitro BBB-on-chip models serve as a platform to investigate cancer metastasis of the brain along with the possibility of efficiently screening chemotherapeutic agents against brain malignancies. In order to improve the proficiency of BBB-on-chip models, hydrogels have been widely explored due to their unique physical and chemical properties, which mimic the three-dimensional (3D) micro architecture of tissues. Hydrogel-based BBB-on-chip models serves as a stage which is conducive for cell growth and allows the exchange of gases and nutrients and the removal of metabolic wastes between cells and the cell/extra cellular matrix (ECM) interface. Here, we present recent advancements in BBB-on-chip models targeting brain malignancies and examine the utility of hydrogel-based BBB models that could further strengthen the future application of microfluidic devices in oncology research.

## 1. Introduction

The brain is perhaps the most important and metabolically active organ in the body and is known for its dedicated complex structural and functional hierarchy [1]. Although the brain requires a constant metabolic demand, it does not have the capacity to store energy, which makes it susceptible to stern consequences even in the case of trivial changes [2]. Therefore, the supply of oxygen and blood to the brain is vital for its precise functioning, with this supply being regulated by a group of cells described as the neurovascular unit (NVU) [3]. The concept of the NVU was provided by Harder in 2002 and describes a structure that combines the neural and vascular components of the brain which regulate the physiology of the brain [4]. An important component of the NVU is the blood–brain barrier (BBB), which represents the blood–brain interface that separates the brain from the systemic circulation. The BBB is a multifarious entity that mediates communication between the central nervous system (CNS) and the periphery and is comprised of the structural, metabolic, and transport elements [5,6]. The BBB not only serves as a physical barrier but also acts as a metabolic barricade, a transport interface, and a secretory body [7].

The BBB plays a crucial role in maintaining the internal milieu inside the brain by supervising the constitution of cerebral extracellular fluid (ECF) and imparting protection against the invasion of harmful agents [8]. The essential constituents of the BBB include endothelial cells (ECs), astrocytes (ACs), and pericytes (PCs) (the most profuse type of cells in the brain). The EC of brain capillaries serves as the first line of defense against foreign circulating bodies, whereas ACs are credited as a metabolic sensor for fluctuations in the brain microenvironment [9,10]. The disruption in the integral structure of the BBB is a common sight in brain tumor (glial tumors, medulloblastomas, and brain metastases) progression, hence the term blood–tumor barrier (BTB) [11].

Although the BBB represents an essential barricade for the purpose of brain protection, it also serves as a barrier to therapeutic interventions during brain tumor progression, and because of this a number of chemotherapeutic drugs such as vincristine, paclitaxel, etc., are unable to reach the brain [12]. Hence, effective BBB models are required to investigate the microenvironment of the NVU and develop a distinct understanding of the behavior of the BBB, which is vital when designing efficient strategies to treat brain tumors. In this regard, tumor-induced animal models have provided important information on the various aspects of the BBB; however, such findings cannot be fully translated to the human platform [13]. Also, the complicated physiology of animal models makes it difficult to perform studies concerning BBB interference at cellular levels in real time [14]. Owing to these challenges, and in order to successfully achieve the clinical translation of developed therapeutic interventions, it is imperative to develop an effective in vitro model that can imitate the complete anatomical, physiological, structural, and functional aspects of the BBB, along with their interactions with therapeutic molecules, in real time.

**Figure 1:**
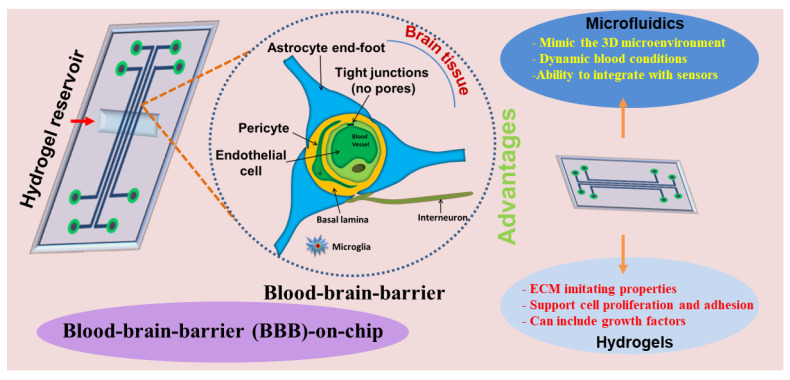
Overview of the hydrogel-based BBB microfluidic model.

Conventional two-dimensional (2D) in vitro models such as the Transwell system have extensively been used for studying the basic features of the BBB, such as barrier permeability and transepithelial/transendothelial electrical resistance (TEER) [15,16]. However, these traditional models lack dynamic blood flow conditions, and are thus not able to recapitulate the dynamic microenvironment of the BBB. To overcome these limitations, microfluidic-based 3D in vitro BBB models have recently attracted the attention of many researchers due to their ability to precisely recapitulate the physiological aspects of the BBB [17,18]. Microfluidic-based models utilize biomaterials, cell components, and growth factors to study and analyze the capability of the designed BBB models in terms of real time investigation. Among several biomaterials available, hydrogels are a persuasive candidate due to their flexibility, ease in terms of chemical modification, and versatile characteristics (Figure 1).

In recent years, many researchers have compiled experimental outcomes in this field with outstanding synopses. The review by Augustine et al. presents a design concept for bioengineered microfluidic-based BBB models in the field of oncology [19]. Moreover, the review by Ahn et al. mainly focused on the understanding of the transport mechanisms of nanoparticles through microengineered human BBB models [20]. Another review by Oddo et al. provides an overview of the recent developments and challenges associated with designing microfluidic-based in vitro models for the BBB [21]. The review by Jiang et al. discussed the technical and operational details of microfluidic model design, fabrication, evaluation, and application [22]. However, there has been no dedicated review that analyzes and critically evaluates the role of hydrogels in designing microfluidic in vitro BBB models for oncology research.

Complementing the aforementioned reviews, in this review, the salient features and physiology of the BBB and its dysfunction/dysregulation upon tumor invasion is discussed. Further, the review aims to explore the salient features of hydrogels that can be explored to recapitulate the architecture of the BBB and its microenvironment. The review also highlights the practicalities and attributes of various microfluidic-based BBB designs and their applicability for oncology research (brain primary tumor and metastasis). In this review, we discuss the available hydrogel-based (natural and synthetic) microfluidic BBB models and their salient features. Finally, we present some emerging challenges that accompany hydrogel-based microfluidic BBB models along with the future prospects of this fascinating area. Due to the emergent status of the field of hydrogel-based microfluidic BBB models dedicated to oncology research, application-oriented literature reported over the last five years was the primary focus in this review. The overall aim of this review was to offer readers a better understanding on hydrogel-based BBB microfluidic devices for brain malignancies.

## 2. Blood–Brain Barrier (BBB)

### 2.1. Salient Features of the BBB

The BBB, located in the brain capillaries, plays a strategic role in maintaining the homeostasis of the CNS and protecting it from detrimental factors and pathogens present in the blood. Early electron microscopic studies on the BBB claimed it to be mainly composed of specialized ECs lining the blood vessels that stream through the brain [23], representing almost 370 miles (595 km) of exchange length amid brain and blood [24]. These ECs are interconnected to each other through tight junctions (TJs) which also serve the function of obstructing paracellular spaces. The microvascular ECs of the brain are extremely thin (39% less thick) in nature when compared to muscle ECs [25]. Also, the ECs present in the brain differ from the ECs that exist in the peripheral tissues in that they exhibit limited endocytotic vesicles, facilitating circumscribed transcellular flux, and the presence of TJs, which restricts paracellular flux [26,27]. The main constituents of TJs are transmembrane proteins such as claudin and occluding-1, -3, and -5, along with the junctional-associated molecule (JAM), in close association with the cytoplasmic scaffolding proteins zonula occludens 1, 2, and 3 (ZO1, ZO2, and ZO3). Furthermore, the BBB ECs also express specific receptors, enzymes, and efflux pumps which helps in the supplying of essential nutrients to the CNS and the removal of waste materials [28,29].

Due to the presence of TJs within the brain capillary endothelium, intercellular pores are absent to prevent brain endothelium with minimal fluid-phase pinocytosis [30]. The presence of intracellular pores is a characteristic feature of EC-based barriers in other peripheral organs. The lack of paracellular space within the BBB allows the brain ISF (interstitial fluid) to access to circulating molecules via two mechanisms, with the first one being lipid-mediated free diffusion through the BBB and the second one being via receptor-mediated transport (RMT) across the BBB [31,32]. Two main classes of transporters are expressed by BBB ECs and include efflux transporters (ET) and nutrient transporters (NT). The ET are differentiated to the lumenal surface to regulate the transportation of a wide range of lipophilic molecules which otherwise are diffused toward the blood [33]. The fundamental efflux transporters that aid in the movement of molecules to and fro across the BBB include: P-glycoprotein, breast cancer resistance protein, BBB choline transport, glucose transporter, and brain multidrug-resistance protein [34,35]. Moreover, the NT expedite the transportation of required nutrients (glucose, lactate, pyruvate, etc.) into the CNS along with the removal of waste materials from the CNS into the blood [36]. Furthermore, the transport of macromolecules (insulin, albumin, transferrin, etc.) across the BBB involves receptor-mediated transport, adsorption-mediated transcytosis, and active efflux transport [37].

**Figure 2:**
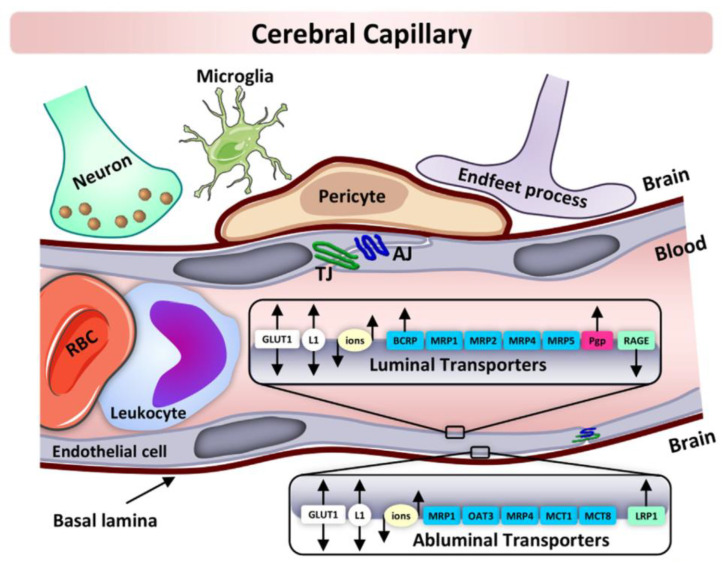
A detailed overview of the cellular constituents of the BBB. Reprinted with permission from Ref. [47]. Copyright 2021 Elsevier.

The brain ECs are further supported by a discontinuous layer of perivascular cells, mainly PCs and ACs. These perivascular cells perform a significant role in the formation and functioning of the BBB [38]. The properties of the BBB are mostly expressed within the ECs but are influenced and sustained through inter and intra communication with mesenchymal-like PCs, ACs, immune cells, and neural cells. The PCs rests on the ablumenal surface of the microvascular endothelium and are rooted in the vascular basal membrane [39]. PCs are often confused with other cells in the perivascular space due to the lack of specific markers expressed uniquely by PCs [40]. They play a key role in extending long cellular processes and are capable of controlling the dimeter of capillaries due to their association with contractile proteins [41]. Apart from regulating the formation of the BBB, PCs also participate in regulating angiogenesis, immune cell infiltration, and blood flow, along with other key roles in wound healing and ECM deposition [42]. Furthermore, in the context of the astrocytic endfeet process, the ECs of the BBB have a strong association with ACs, which are positioned at a tactically significant location between the endothelial blood flux and neurons and play a vital role in the regulation, formation, and maintenance of the BBB [43]. There is growing evidence that ACs are involved in the upregulation of many BBB features, including the tightening of TJs [44,45,46]. Figure 2 gives an overview of the cellular constituents of the blood brain barrier [47].

The infiltration of cells into the CNS is highly restricted by the BBB, but preferential metastasis of some cancer to the brain is very surprising; hence, it would not be an overstatement to link the role of the BBB to the support of metastasis formation. As metastatic cells relish protection against immune scrutiny, it is anticipated that some releasing factors from the cellular components of the BBB may favor metastatic growth.

### 2.2. Role of BBB in Oncology

New therapeutic interventions have augmented the treatment possibilities for cancer in most parts of the body, but the efficacy of these new clinical modalities is still unrealized in case of primary or secondary (metastatic) brain tumors [48]. Although every tumor type has the capacity to metastasize towards the brain, melanoma, lung, and breast cancer have the highest metastatic rates, with lung and breast cancer contributing to more than 75% of all secondary brain tumors [49]. An effectual tumor treatment experiences many impediments, of which multidrug résistancy (MDR) is an imperative aspect. MDR can be defined as the ability to develop resistance (intrinsic or acquired) towards a wide spectrum of chemotherapeutic drugs with diverse cellular targets and chemical structures [50,51]. In general, tumors consist of both drug-sensitive as well as drug-resistant cells. Chemotherapy drugs usually kill the drug-sensitive cells but allow a significant number of drug-resistance cells to proliferate. As the tumor grows, chemotherapy becomes ineffective, as the remaining tumor cells consist mainly of drug-resistant cells [52]. The resistance that develops in tumors is mainly associated with molecular pumps present on tumor-cell membranes which enable tumors to expel chemotherapy drugs.

To date, mRNA for nearly 15 drug transporters have been identified in brain capillaries, which include but is not limited to p-glycoprotein (Pgp), multidrug resistance-associated protein (MRP), multidrug-resistance protein, organic anion transporting polypeptide (OATP), and organic anion transporter (OAT) [53]. In context of the BBB, Pgp has gained considerable attention, as it has been shown to be an integral part of the BBB. Reported research findings suggest that the expression of Pgp in the ECs of the BBB can restrict the entry of certain drugs into the CNS [54,55,56]. Since Pgp is actively involved in the efflux of many hydrophobic drugs out of the cell, it is anticipated that Pgp might stand accountable for the poor infiltration of large hydrophobic drugs (>400 Da) in the brain through the active back-transport of these drugs to the blood [57]. The breakdown of the BBB is a common occurrence in high-grade primary brain tumors and brain metastases, resulting in the formation of the BTB, which offers heterogeneous permeability to many chemotherapeutic drugs, influencing their poor therapeutic efficacy. However, the proficiency of the BTB is more common in cases of low-grade gliomas and at the invasive boundary of high-grade brain tumors [58,59]. A key factor associated with the BBB, especially in the case of high-grade brain tumors, is the tumor-secreted vascular endothelial growth factor (VEGF), the production of which is accelerated by the persistence of the hypoxia condition in the tumor microenvironment [60]. The secreted VEGF then stimulates disruption in the structural architecture of the BBB and encourages angiogenesis via the reinforcing of the development of abnormal capillaries [61,62]. In order to address the enhanced metabolic obligations of the growing tumor cells, the developed vascular endothelium exhibits the expression of anomalous outlines for the related transporters and receptors [63].

Changes in the structural architecture of the BBB upon tumor invasion are influenced by the tumor stage, volume, type, and location [59]. The gravity and extend to which the BBB is impaired ranges from critical damage, in case of brain gliomas, to minor destruction, in case of other pathogenic conditions such as strokes, neurodegenerative disease, and obesity [64,65]. The function and the structural architecture of the BTB for low grade gliomas is comparable to the normal BBB, but in case of high-grade gliomas, the vascular thickness, microvascular blood volume, and surface area of the BTB are substantially higher [66,67]. It is observed that the degree of invasion of the brain by the tumor cells is independent to the level of damage experienced by the BBB. Interestingly, an intact BBB may protect an infiltrating glioma cell from therapeutic interventions. On the other hand, with the infiltration of a glioma cell, the foot process of ACs can push away from the vascular endothelium, which in turn leads to localized fracture in the BBB [68,69].

Although the intrinsic architecture of the BTB is disrupted inside the tumor, there are reports of BTBs retaining intact features in some specific areas in a tumor [59,70]. These features reflect the heterogenic character of the BTB, with it comprising of existing and newly formed blood vessels that supply nutrition to the glioma. Thus, it is evident that the BBB plays a major role in regulating primary and secondary tumors and also plays a key role in restraining the efficacy of chemotherapeutic drugs.

### 2.3. Considerations to Recapitulate BBB Models

Disruption or alteration in BBB architecture is associated with many pathological conditions, but the severity of its disruption is mainly connected to gliomas [71]. Hence, establishing an appropriate model that comprehensively imitates the biophysical nature of the BBB is required to appreciate the clinical signatures of the BBB linked with specific ailments and also to understand its architectural features along with its pathophysiological attributes. Considering the architecture of the BBB, which is comprised of neural and vascular components along with multiple dynamic proteins, creating a BBB model capable of recreating that complexity has been an arduous assignment. Over the passage of time, a wide range of models (in vitro, in vivo, in situ, ex vivo) have been proposed to replicate the BBB [72]. The selection of any specific model depends on the downstream application targeted by the researchers.

Although establishing a BBB model is a challenging task, a successful model should meet specific requirements and parameters to guarantee data consistency, reproducibility, and precise correspondence with the complex nature of the BBB. Any established model of the BBB should accurately mimic the BBB architecture, including ECs, Acs, PCs with luminal and abluminal sides, and articulate TJs and adherence junctions (AJs) in the endothelium [47,73]. The reliability of the model will also depend on its ability to proliferate and differentiate ECs while including glia and apical membrane. Moreover, the established model should have coherence in ion pairing and selective permeability towards the transportation of ions such as sodium (Na) and potassium (K) with negligible paracellular diffusion across ECs [72]. In order to mimic the BBB, the model should be able to express different uptake and efflux pumps along with high activity of BBB-linked enzymes such as gamma-glutamyltranspeptidase, monoamine oxidase, alkaline phosphatase, etc. 

While recapitulating the anatomy and physiology of the BBB, genetic heterogeneity could be a vital factor. Clinical translations of many drugs for human use are unlikely, even they demonstrate commendable therapeutic effects during animal testing. This could be due to the difference in the genetic constituents of animal models and humans, which restricts the complete reproduction of an organoid/tissue. Hence, human-originated cells would definitely have an edge in the design of BBB models. Concerning the permeability attributes, a model should have selective permeability for different therapeutic molecules based on their molecular weights and inherent lipophilic nature. Moreover, the model should respond to change in terms of the permeability of the barrier upon interaction with external agents (enhancers) and encounters with pathological conditions such as diabetes and hypertension. Preferably, the established model should allow for the real time assessment of different cellular level interactions, permeability, and imaging and should be dynamic in nature. Overall, apart from the aforesaid benchmarks, the model should be straightforward and effective in terms of cost, reliable, easy to handle, reproducible, and experiment friendly.

## 3. Microfluidic-Based BBB Platform

### 3.1. Overview

Establishing a realistic BBB model could have widespread applicability in studying the transportation dynamics across BBBs under normal circumstances and diseased condition and aid in the development of efficient therapeutic interventions with passable distribution in the brain. Scientific communities have invested a lot of effort in the development of in vitro BBB models to avoid the difficulties faced in the clinical translation of results obtained from animal models. In this regard, several 2-dimensional (2D) in vitro models have been established with mono/co/tri culturing of human derived cells (ECs/PCs/ACs) in order to imitate the transportation pattern of different molecules across BBB endothelium [74,75,76,77]. These methods feature progressive refinement aids to promote the differentiation of human pluripotent stem cells (hPSCs) into brain microvascular ECs and display the identified attributes (the uptake of lipophilic molecules, high TEER, and transporters activities). However, these models lack the ability to mimic the 3D microenvironment and cellular organization of the anatomical and physiology architecture of the BBB [78]. Recently, a great emphasis has been placed on the development of microfluidic-based 3D models in which the relevant cells are implanted in a physiologically significant gel matrix. Microfluidic-based BBB models provide compelling prospects in regulating and studying the mass transport of therapeutic and signaling molecules, active biological agents, and nutrients for real time biological analyses [79,80].

The term microfluidics is defined as the study of systems comprising miniature quantity of fluids at the submilliliter dimension. Microfluidic technology involves the designing of miniature systems and studies involving physics, performance, and control of fluids, confined in small channels [81,82]. The technology of microfluidics is utilized to evaluate a number of consequences that are difficult to be analyzed and monitored at high volume fluid systems. This field is of significant importance in the health care sector where miniature fluidic system comprising of chambers and channels is used for processing liquids, separating relevant molecules, detection of molecules under investigation and analysis of individual components and factors [83]. Microfluidic devices are associated with certain attributes which includes high sensitivity and specificity, controlled fluid handling, minimum amount of sample requirement, cost-effectiveness [84].

In 1990, Manz et al. created the term “miniaturized total chemical analysis systems (µTAS)” to describe small-volume related reactions [85]. Subsequently, with the evolution of technology, the more comprehensive term “microfluidics” replaced the term µTAS, followed by the concept of organ-on-a-chip (OOC), also referred as tissue-chips or organ-chips, which refers to microfluidic multichannel devices used for culturing biological cells in miniature spaces to replicate complex multicellular tissues and organs. These microfluidic devices are capable of recapitulating the 3D niche of the organ/tissue under investigation and establishing realistic responses and activities when compared to human organs [86,87]. OOC technology precisely regulates the factors that directly influence the performance of in vitro experiments resulting in reliable, reproducible, and accurate results [88].

The first practical model of OCC-based microfluidic chips was fabricated in 2010, when Donald E. Ingber’s research team at the Wyss Institute at Harvard University first demonstrated a practical model of a lung-on-a-chip [89]. Subsequently, remarkable progress has been made in the field of OOC technology for different human organoids and tissues, with precise applications, including drug testing and screening, understanding the pathophysiology of diseases, and real time cellular analysis [90]. These engineered microfluidic devices provide a platform for the design of new drugs, screening of biological active agents for novel therapeutic interventions, and understanding possible pathways that are involved in the comprehensive spread of any pathological condition.

In order to imitate the anatomical and physiological aspects of the BBB, an ideal in vitro BBB model should able to address some key attributes of in vivo BBBs which include: intercellular interactions, the vessel-like structural architecture of ECs resembling 3D vessels, flow dynamics provoked by shear stress on ECs, and the selective permeable basal membrane (BM) [91]. Further, the recapitulation of intact BBBs on microfluidic chips is influenced by many factors including different designs, the source of ECs, and the chip fabricating materials.

### 3.2. Design Concepts of Microfluidic BBB Models

**Figure 3:**
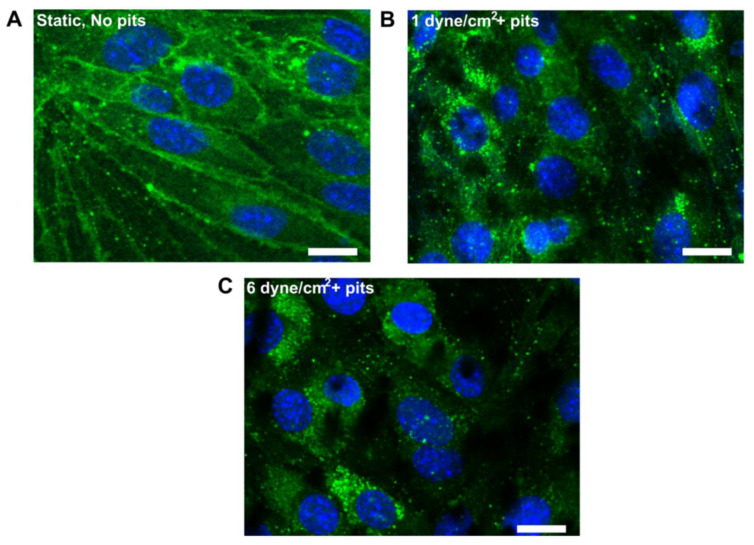
Characterization of microfluidic BBB model function. (**A**) Claudin-5 staining of brain EC cultured for 6 days in static cell culture plates. (**B**,**C**) Claudin-5 staining after 6 days static culture followed by 2 h flow. Scale bars are ~10 µm [94].

Microfluidic platforms evolved from the orthodox Transwell design, which comprises of porous membranes placed between upper and lower channels to create a sandwich-like assembly. Here, the two chambers define neural and vascular channels separated by a porous membrane. The complete system represents vascular channels and the complete architecture of an intact in vivo BBB [92]. In this sandwich design, the ECs are generally cultured in the upper channel, whereas the lower channel is the platform for seeding other brain cells such as PCs and ACs, etc. The involvement of two microchannels stimulates the passage of the culture medium, representing the dynamic nature of circulating blood and ECM [93]. Polycarbonate-based membranes have been widely utilized in designing microfluidic BBB models. In a study by Papademetriou et al., a microfluidic device comprising two S-shaped microchannels were placed vertically and separated using polycarbonate membrane [94]. The work studied the effect of the local flow environment on the transportation and penetration of Angiopep-2 (peptide functioning as brain transport vector) with liposomes. In this study, the distribution of TJ proteins following the culture of brain ECs on the substrate and acute exposure to fluid shear stress expression was studied using analysis of the expression of Claudin-5, the most enriched TJ protein. It was revealed that claudin-5 appeared primarily perinuclear in brain EC cultures after 2 h of flow exposure. This observation suggested slight damage of the TJ during the flow exposure, which could influence the diffusion of liposomes across the BBB model (Figure 3).

In another study, Booth et al. designed a multi-layered microfluidic device comprised of two vertical microchannels separated by porous polycarbonate membrane along with multiple embedded electrodes [95]. In order to ensure laminar flow, the channel dimensions were kept at 200 μm (height), with 2 mm (luminal) or 5 mm (abluminal). The validity of the designed microfluidic system was compared with many reported models and offered enhance permeability. The use of polycarbonate-based membranes are often associated with certain limitations, which include poor transparency, high resolution image capture, and cell culturing arrangements that restrict the real-time assessment of cell growth and molecular transport [96]. These shortcomings could partially be addressed with the use of polytetrafluoroethylene-based membranes, which were also reported to impart additional stability to sandwich designs [97].

**Figure 4:**
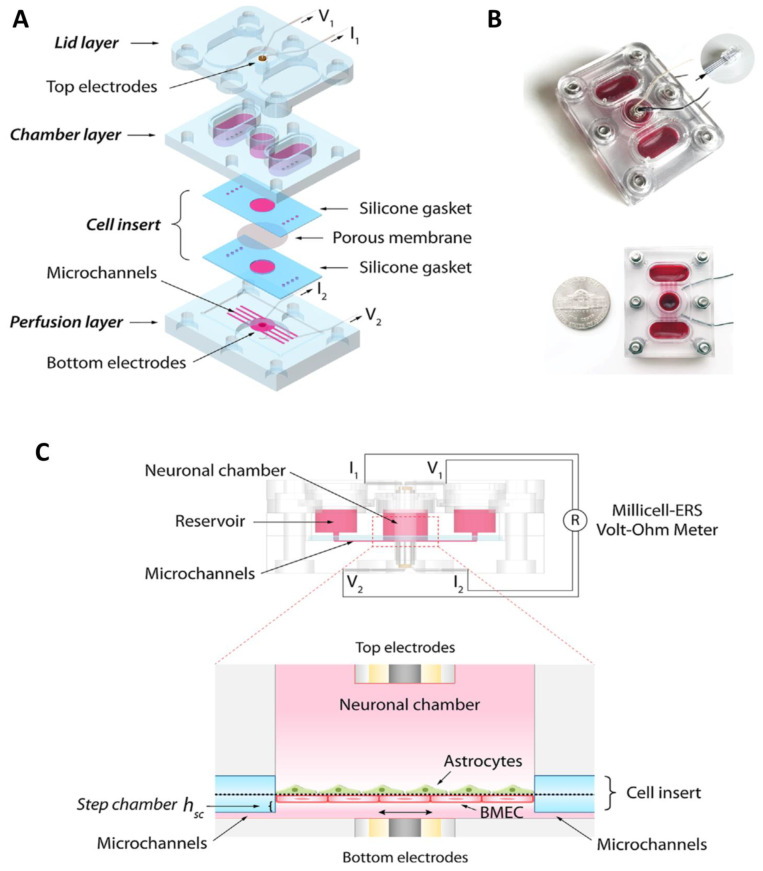
Design of the BBB-on-a-Chip. (**A**) Schematic exploded view of the microfluidic platform. (**B**) The assembled device, with or without the lid. In order to visualize the microfluidic device (microchannels, neuronal chambers, reservoirs), a red dye was used. (**C**) Side view showing the fluid pathway, electrode wiring, and the BBB co-cultural orientation. The zoom-in panel showing the cross-sectional view. Reprinted with permission from Ref. [100]. Copyright 2017 John Wiley and Sons.

Moreover, the examination of cell behavior still remains a challenge, which could be confronted through the use of parallel designs in which the culture chambers are arranged horizontally and separated with polydimethylsiloxane (PDMS) membrane. The advantage of parallel designs is their utility for high resolution image capture due to improved observations of cell behavior [98]. The use of PDMS membrane not only imparts transparency, but it also features much larger pores and thicker barrier features compared to natural BBB [99]. Wang et al. proposed a parallel designed microfluidic BBB model that provides in vivo-like characteristics and is applicable for screening drugs for their permeability [100]. The design consists of three layers, with the first layer consisting of a chamber layer holding two reservoirs and a neuronal chamber at the center. The second layer is a medium perfusion layer with a pocket housing human brain microvascular ECs (BMECs) and ACs, with microchannels linking the luminal chamber with the reservoirs. The third layer (lid layer) covers the reservoirs and the neuronal chamber to permit gas exchange with minimal medium evaporation (Figure 4). This designed platform could be implemented for integration with other organs to imitate multi-organ interactions in response to drugs. Several parallel-designed microfluidic devices have been reported with diverse functions and applicability.

PDMS-based microfluidic BBB models are generally fabricated with rectangular cross-sections in the microchannels, which may result in non-uniform shear stress and profiles. These uneven flow dynamics may cause divergences in the behavior and morphological aspect of the seeded ECM along the channel [97]. To overcome these limitations, cylindrical-shaped microchannels can be constructed, through which a constant shear stress can be maintained along the entire inner walls. These modifications result in the formation of a 3D tubular structure design. However, a crucial limitation of such a design construct is its impediment to measuring barrier tightness owing to its cylindrical design [98]. While majority of the BBB device constructs emphasize the arrangement of microvessels in relation to the fabricated microchannels, a less explored strategy, the vasculogenesis strategy, has also been practiced to construct microvessels via de novo formation from endothelial progenitor cells. Generally, in the case of the vasculogenesis strategy-based devices, the ECs are co-cultured with either fibroblasts or mesenchymal stem cells (MSCs) within a perfusable microchamber comprised of an ECM imitating the 3D environment [101].

As an advanced technology, microfluidics-based devices have been able to effectively recapitulate complex and 3D microenvironments of tissues with suitable regulations. Thus, as an alternative, microfluidic devices have been successful in overcoming and limiting the use of animal models for studying the physiology and function of tissues/organs pre/post pathological conditions. This reputation has been propelled due to the incredible progress seen in microfabrication and microfluidic techniques, which have been influenced by salient BBB-specific features which need to be examined for model integrity. A summary of the existing BBB-on-chip models for oncology research is presented in Table 1.

### 3.3. Characteristics of Hydrogels for BBB-on-a-Chip Platform

**Figure 5:**
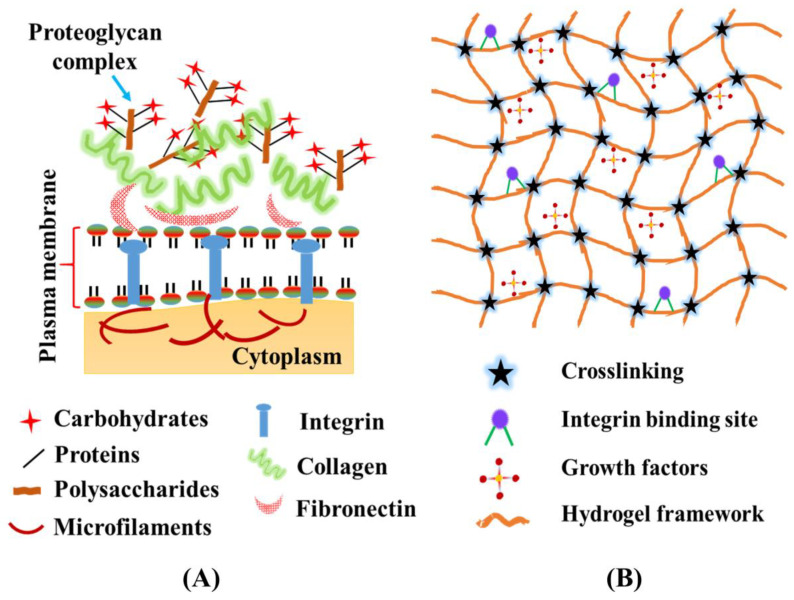
(**A**) Components of extra cellular matrix (ECM); (**B**) structural framework of hydrogels imitating the biophysical and functional prospective of ECM.

Hydrogels are a category of 3D crosslinked polymeric network structures capable of imbibing a large amount of water. Owing to their tunable properties and versatile fabrication methods, hydrogels have been applied in numerous biomedical applications ranging from drug delivery to tissue engineering and regenerative medicine [112]. In order to recapitulate the 3D microenvironment conducive for cell growth, hydrogel-based scaffolds have been explored due to their unique physical and chemical properties, which mimic the 3D micro architecture of the tissues [113]. In order to adhere and proliferate cells, the hydration and porosity of hydrogels are very crucial in providing the necessary growth factors and a platform that maintains osmotic pressure and normal cell functionality [19,114]. In terms of microfluidic platforms, hydrogels have been used as a material of choice to imitate native ECM features (Figure 5). Hydrogels allow for the exchange of gases and nutrients and the removal of metabolic wastes between cells and the cell/ECM interface, and thus are able to regulate ECM behavior. This, in turn, promotes a sequence of cellular activity in 3D models, which includes cell proliferation, adhesion, differentiation, migration, and cell–cell/cell–matrix interactions [115,116].

When selecting hydrogels as an ECM-imitating material for microfluidic-based BBB models, the innate properties of hydrogels are very important. The mechanical features of the ECM memetic hydrogel material, such as its stiffness, elasticity, stress, strain, and porosity, play a significant role in cell adherence, migration, and cytoskeletal assembly. In general, rigid ECM materials may possess a higher stiffness than that of brain microvessels [117,118]. The mechanical attributes of the hydrogels are mainly evaluated in terms of elastic modulus (EM) and shear modulus using rheology. The elastic modules of material used to mimic the ECM for BBB architecture should have an EM in the range of >1 KPa, close to that of the brain parenchyma [119,120]. Moreover, porosity plays a significant role in the cell culture while modelling the BBB, as the pore size can influence the perfusion of nutrients, growth factors, and gaseous exchange in the hydrogel network [121]. An appropriate porosity for the hydrogel scaffold is required to achieve the desired cell proliferation, migration, and differentiation, as large pores hinder cell–cell interactions [122]. The distribution of pore sizes across hydrogels while imitating the ECM is very crucial, as smaller pore sizes can restrain the migration of cells toward the center, dissemination of nutrients, and elimination of waste materials from the hydrogel network, and larger pore sizes can reduce the total surface area pertaining to cell adherence [123]. In addition, the selection of a crosslinking agent during hydrogel fabrication is also vital, as some of the crosslinking agents used for hydrogel fabrication can also have detrimental effects on cell proliferation and viability [124].

Another important aspect of hydrogels that needs to be considered when selected them in the design of a microfluidic-based model is their degradation properties. The degradation of hydrogels over time can lead to alterations in their mechanics and swelling properties, which may affect cellular activities such as motility, migration, and traction force generation [125,126]. While the degradation mechanism of hydrogels can be accounted for using either hydrolytic or enzymatic means, in terms of BBB microfluidic models, more emphasis is given to the hydrolysis aspect of degradation. Hydrolysis in hydrogels transpires due to the existence of unstable chemical bonds within the framework of the hydrogel architecture. The rate of degradation in the case of hydrogels can be fine-tuned via changes in the polymer concentration, crosslinking density, and crosslinking rate during the fabrication process [127]. Moreover, technological advancements in the field of polymer chemistry have inculcated external triggered responses (ultraviolet, infrared, etc.) to tackle the degradation mechanism [128]. It is important to note that the property of degradation is a relative term, as the hydrogels that fall under the category of non-degradable behave invariably during the course of experimentation; however, they may eventually be degraded. It is the stability of hydrogels during the time frame of studies that makes them non-degradable. When designing hydrogel-based microfluidic models, the sterility of hydrogels before their direct interaction with cells is also of prime importance. There are a number of ways that hydrogels can be sterilized, which include but are not limited to the use of UV/gamma irradiation, ethanoic solution, and supercritical carbon dioxide, etc. [129,130]. All these approaches are effective but should be chosen wisely, as the applied process should not degrade the hydrogel, denature the active components, or make alterations to the innate properties of the hydrogel.

Hydrogel-based 3D BBB-on-chip models mainly depend on the injection of hydrogels into a central compartment which is surrounded by two peripheral fluidic channels. In these external channels, ECs are seeded to establish adherence with the hydrogel in order to stimulate the vascularization of the matrix [131,132]. The use of hydrogels for BBB models is not limited to mechanical features for recapitulating the ECM, but has been reinforced by the introduction of conducting polymers, resulting a new class of functional hydrogel that not only offers the opportunity to replicate the physical properties of the ECM across BBB but also mimics its electrical conductivity [133,134]. Hydrogels have proven to be potent candidates in various biomedical applications. The next section will concentration on different studies based on hydrogels that have been used for 3D microfluidic BBB models for the purpose of oncology research.

## 4. Hydrogel-Based Microfluidic Models in Oncology Research

Microfluidic BBB models are micrometre-sized mechanical channels comprised of 3D arrangements of multiple cell types and a hydrogel matrix to mimic BBB complexity. These platforms offer more resemblance to the brain microvasculature and its physical and biological microenvironments than the traditional in vitro BBB platform. The model is comprised of four main components, including a hydrogel 3D matrix, a continuous supply of fluid with shear stress and controlled osmotic pressure, a brain tissue chamber, and an array of biosensors for the long-term monitoring of the BBB microenvironment [19]. The hydrogel matrix in BBB models is an integral component when it comes to providing microchannels and spatial orientation to mimic a 3D analogy of the native ECM. This analogy allows for improved cellular communication, deliberate cell attachment sites for the bioactivation of the cellular factors required for cell proliferation, differentiation, and migration in the in-vitro cultures. These devices demonstrate the high retention and rapid growth of ex-vivo grown cells, facilitate the smooth and homogenous distribution of nutrients, and maintain the homogeneity of the cultures along with providing the real-time microscopic monitoring of physiologically relevant shear conditions for the ex-vivo growth of cells [135].

The importance of hydrogel matrices in microfluidic models has grown substantially in recent decades because of their capability to form complex networks for providing a microenvironment for cellular growth and their flexibility to modulate as per the application requirements [136,137]. The choice of hydrogel matrix in the microfluidic device is highly dependent on the application of the device. Natural polymer-based hydrogels matrices secrete endogenous factors which endogenously support cellular attachment and growth. These polymers are typically derived from ECM proteins including collagen, fibrin, and hyaluronic acid or natural sources such as chitosan and alginate [124]. However, their low mechanical and biochemical strength, high degradability, the potential of contamination, and batch-to-batch variability in mass production raised concerns about the sensitivity and durability of natural hydrogel matrices in microfluidic based model systems. These limitations were overcome by the development of synthetic hydrogels, which provide flexibility in terms of chemical modification, varying degrees of porosity and stiffness, improved stability, biocompatibility, degradability, and the tuning of the mechanical strength for different cellular applications. Commonly used synthetic hydrogels in microfluidics are poly-ethylene glycol, poly-vinyl alcohol, polyacrylamide, poly-aspartic acid, and poly-2-hydroxy ethyl methacrylate [138,139]. These hydrogel-based 3D microfluidic cell culture devices can be used to investigate cell proliferation, metastasis, cell-to-cell contact as well as in vitro drug screening in oncology research. A brief description of hydrogel-based BBB models in oncology research is discussed in the following sections.

### 4.1. Natural Hydrogel-Based Microfluidic BBB Models

Glioblastoma (GBM) is a highly aggressive primary brain tumor, with a median survival of <15 months [140]. Despite technological advancements in the field of medicine, even after surgical removal, followed by radiotherapy and chemotherapy, complete control over the ailment is still unaccomplished [141]. In order to study the origin of GBM and its progression, a microfluidic device was developed by Chonan et al. using HUVECs and type I collagen [142]. The device mimics the 3D brain tumor microenvironments and showed the HUVEC-induced migration of nestin-positive tumor cells into type I collagen gel and that genes such as integrin α2 and β3, which are associated with migration or metastasis, were significantly upregulated. These results provided valuable inputs for the development of effective therapeutic strategies for GBM. The study revealed that the invasion induced by HUVECs was primarily led by nestin- (neural stem cell marker) positive cells, whereas cells positive for tubulin b3 (TUBB3), a differentiated cell marker, rarely preceded invasion, with HUVECs inducing the upregulation of TUBB3 in GICs.

**Figure 6:**
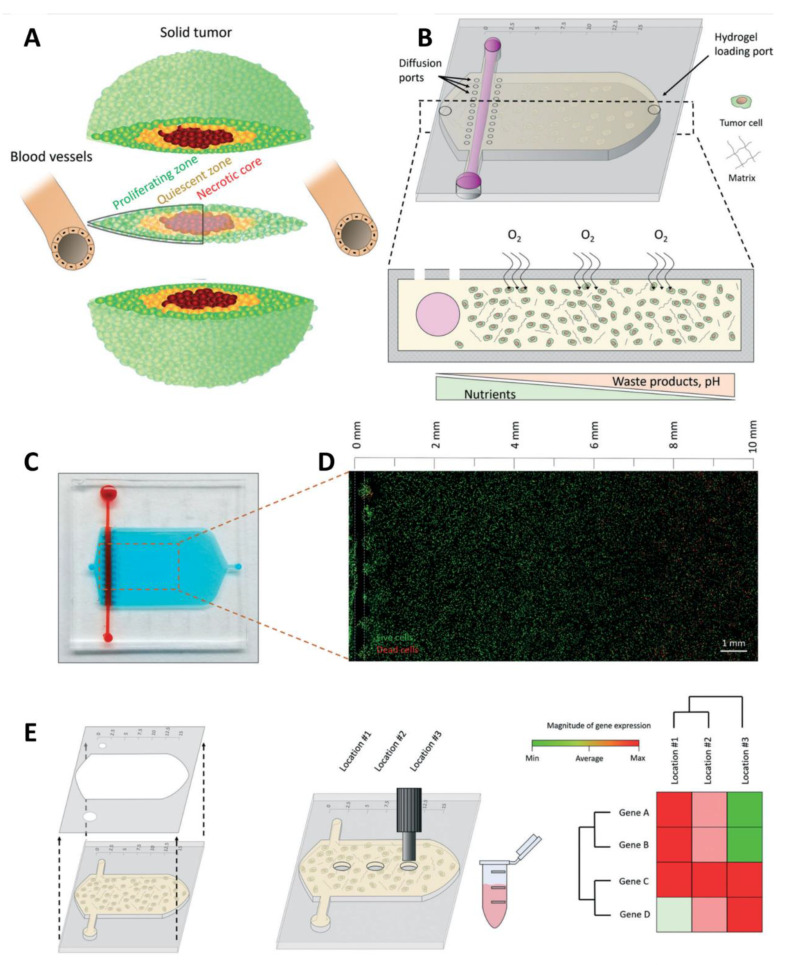
(**A**) Schematic representation of the different tumor phenotypes generated in a solid tumor due to nutrient starvation; (**B**) scheme of the tumor slice microdevice showing the central microchamber, the lumen, and the different loading and diffusion ports. The bottom panel shows the microdevice cross-section. HCT-116 cells were embedded in a collagen hydrogel, the lumen, as well as the pores in the upper half to allow nutrient diffusion. (**C**) Picture of the microdevice filled with blue and red-colored water for visualization purposes. (**D**) Confocal image showing HCT-116 cell viability after 24 h in the microdevice at 10 million cells/mL. Viable and dead cells are shown in green and red, respectively. White dashed line indicates the lumen position. (**E**) Scheme illustrating the protocol to retrieve the cells from the device. Both halves are disassembled, exposing the collagen hydrogel, and then hydrogel punches are isolated using a biopsy puncher. Reprinted with permission from Ref. [143]. Copyright 2019 Royal Society of Chemistry.

Pseudopalisade, a hypercellular zone that typically surrounds necrotic tissue is a characteristic feature of GBM. A custom-designed microfluidic device was developed by Ayuso et al. to imitate the dynamics of pseudopalisade formation in GBM using U-251 MG cells embedded in a collagen hydrogel matrix [143]. In addition, to mimic the vasculature, a lumen was designed on the flank of the chamber through which the media was perfused. The microfluidic model design and operation is presented in Figure 6. Under the regulation of these attributes, tumor cell metabolism led to the production of pH gradients, viability, and proliferation. To mimic the blood vessel obstruction event of the disease, the flow of the medium was controlled through lateral microchannels. In the fabricated device, the two halves could be separated to expose the hydrogel and retrieve the cells after the experiments for downstream analysis. Thus, the designed microfluidic model concluded that nutrient and oxygen starvation triggers a strong migratory process in GBM, leading to the formation of pseudopalisade.

**Figure 7:**
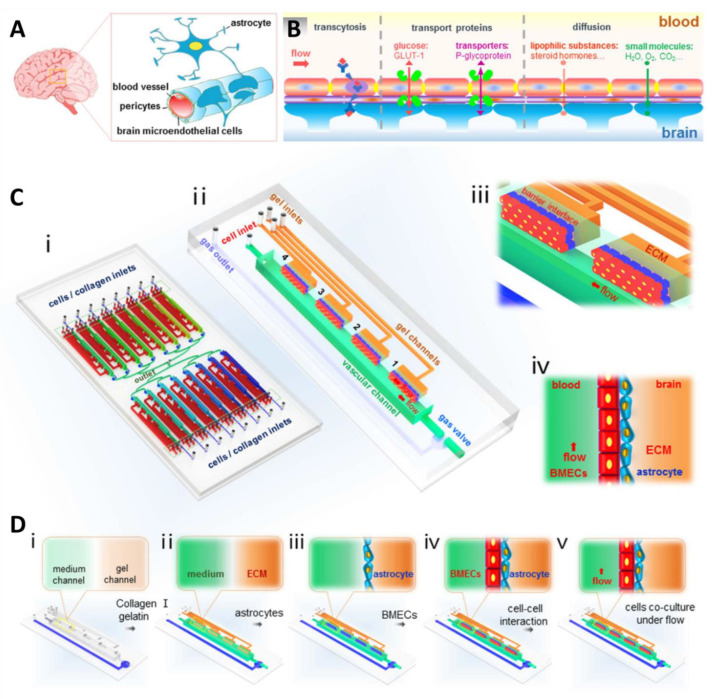
(**A**) Cellular constituents of the BBB in vivo. (**B**) Schematic illustration of BBB function with the expression of several transporters and functional proteins. (**C**) The design and structure of the integrated BBB device: (**i**) device design composed of 16 independent function units connected by a microchannel network (**ii**) consisting of four uniform BBB regions; (**iii**) enlarged view, and (**iv**) side-view of the barrier regions. (**D**) Illustration of the procedures to establish the BBB under flow with the following steps: (**i**) The empty device with closed gas valve and vascular channels; (**ii**) infusion of collagen, gelatin, and cell medium with gas valve opened; (**iii**) suspension of ACs perfused into the vascular channel and attachment to the side surface of ECM; (**iv**) suspension of BMECs perfused into the vascular channel and attached to the ACs; (**v**) co-cultures of BMECs and ACs in the vascular channels under continuous flow [144].

In the field of brain oncology, tumor metastasis is a serious phenomenon that needs considerable attention. An innovative BBB model for metastatic brain tumors could improve our understanding of the condition and help design new treatment strategies. A new dynamic 3D microfluidic system was developed by Xu et al. to replicate the key structural, functional, and mechanical properties of the blood–brain barrier [144]. The microfluidic model is comprised of an array of 16 independent functional units, and each unit has four uniform BBB regions (one vascular channel, one gas channel, one gas valve, and four gel channels) which sharing the same waste outlet. These regions consist of a vascular channel to maintain the flow of fluids into ACs embedded in a natural ECM collagen. The barrier regions of the designed chip consist of BMECs, ACs, and an ECM under flow. The compartmentalized channels allow for the better control of the overflow rate and delivery of nutrients to the cells. This model possesses the unique capability to examine the metastasis potential of cancer cells and their response to chemotherapy (Figure 7). Moreover, Kim et al. [145] developed a 3D model of brain microvasculature using a collagen-based hydrogel matrix with a 3D printing technique. The microchannels used in the model were fabricated from collagen type I using microneedles and a 3D printed frame. The model was used to characterize the barrier function of brain microvasculature by studying transendothelial permeability. It can serve as a useful tool not only for fundamental studies associated with the BBB in physiological and pathological settings but also for pharmaceutical applications.

Recently, Hajal et al. [78] created a self-assembled human BBB model featuring perfusable microvascular networks within microfluidic devices from stem-cell-derived or primary brain ECs and primary brain PCs and ACs. The cells were cultured with fibrinogen solution and thrombin to form a self-assembled hydrogel matrix in microfluidic channels. The model was used to study molecular permeability analyses; however, it also provides robustness and flexibility in numerous other applications, including oncology research.

In an effort to recreate a more physiologically relatable BBB microenvironment with other brain cells types (ACs, PCs, and neurons), various strategies have been explored to introduce ECM-mimicking components. The mechanical attributes and the interstitial flow in the cellular microenvironment influences the propagation of ECs. Also, a high concentration of the selected hydrogel material at the interface ensures the quiescent stage of the endothelium. An ideal hydrogel-based 3D microdevice should ensure an enhanced rate of recovery for a damaged BBB. Efforts are being made to further investigate the use of natural hydrogels as compelling candidates for fabricating 3D BBB models. Also, hydrogel-based BBB microfluidic models could be explored to understand the multicellular interactions across the BBB during the therapeutic intervention process.

### 4.2. Synthetic Hydrogel-Based BBB Models

Synthetic hydrogels are designed to adapt to structural changes in response to various chemical and physical variations. A key aspect of synthetic hydrogels is that they acquire tunable properties along with facile fabrication routes with functional outcomes. Commonly used polymers, as key components for the formation of hydrogels, include polyethylene glycol (PEG), polycarbonate urethane (PU), and poly(epsilon-caprolactone) (PCL) [146]. When using synthetic hydrogels for fabricating microfluidic devices, efforts need to be made to ensure that impurities, by products, an excess of monomers, unaccounted reagents, and catalysts are entirely eliminated before use in order to maintain sterility and biosafety.

**Figure 8:**
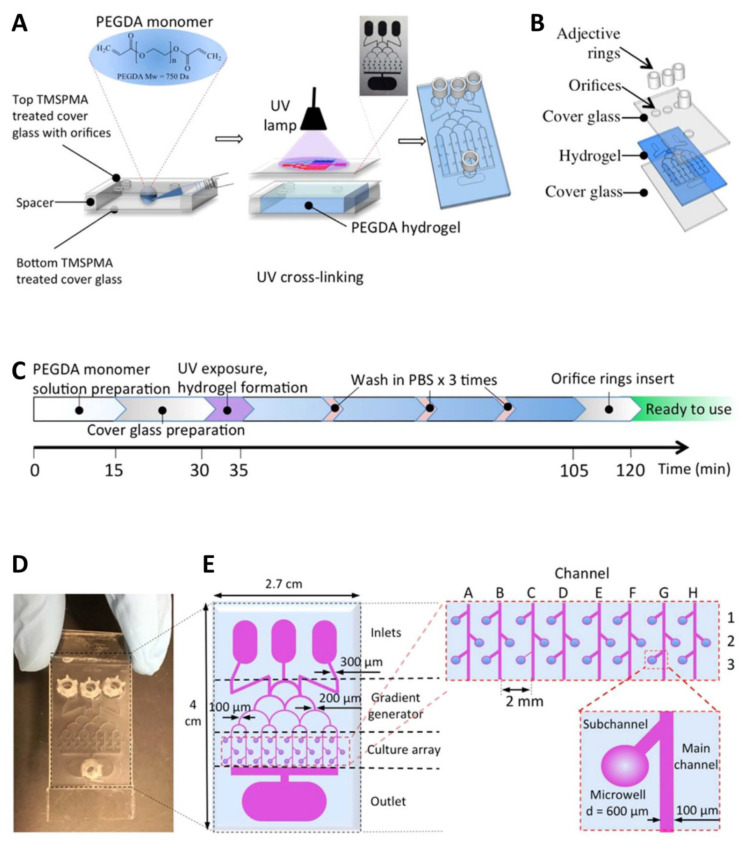
Brain cancer chip design and preparation. (**A**) Final hydrogel device with microchannels and microwells after adding inlet and outlet reservoirs on top of the inlet and outlet orifices. (**B**) Schematic of the layers that are assembled during device fabrication. (**C**) Time protocol of the brain cancer chip preparation. (**D**) Photograph of the device from above. (**E**) Christmas tree-shaped channel system (gradient generator) of the brain cancer chip with channels of gradually decreasing width from 300 μm to 100 μm, an array of 24 individual culture chambers, and three inlet reservoirs and one outlet reservoir. The sub-channels, which link the microwells to the main channel, prevented captured cells from escaping the microwell. Reprinted with permission from Ref. [147]. Copyright 2016 Nature.

In this regard, a novel 3D brain cancer chip model with integrated microfluidic channels was developed using PEG diacrylate (PEGDA) hydrogel and GBM cells (U87) [147]. To study the combinatorial treatment and advantages of pitavastatin and irinotecan, 3D brain cancer tissue was developed from GBM cells in the microwell array. In this study, 3-(Trimethoxysilyl) propyl methacrylate 98% (TMSPMA) was used to create an adhesive facade to coat PEGDA hydrogels on the cover glass (Figure 8). The setup created an ECM-mimicking microenvironment with a massive parallel processing capability along with a tunable transport property. The results showed that the chip is capable of high-throughput GBM cancer spheroid formation and massive parallel testing of drug responses. The fabricated device provides a tunable drug release platform, and administered drugs can be release at desired concentrations over an extended period of time.

To further improve the complexity in terms of the BBB, the cells in the microfluidic system were replaced by patient tissue-derived 3D spheroids. These developed spheroid tissues recapitulate the pathological characteristics and complex ecology of native tumors, meaning that the model can serve as a personalized monitoring device which could help design appropriate cancer treatment for individual patients. The model mimics the biochemical and biophysical properties of the native GBM using a 3D bioprinting process to create a cancer analog on a chip. The device combines a compartmentalized cancer–stroma structure, an oxygen gradient-generating system, and a brain decellularized ECM [111]. The model successfully produced competitive responses to the clinical outcomes of concurrent chemoradiation and temozolomide in GBM. It provides the advantage of being able to determine drug combinations to achieve superior tumor killing and identify effective treatments for glioblastoma patients resistant to the standard first-line treatment. An overview of the different hydrogel-based microfluidic BBB platforms for oncology research is presented in Table 2.

## 5. Conclusions and Future Prospects

The complicated structure and physiological attributes of the brain have always posed challenges when it comes to the transportation of chemotherapeutic molecules across the BBB. The high attrition rate of therapeutic molecules and their constantly mounting mortality rate indicates the ineffectiveness of existing preclinical models in delivering clinically relevant assessments. Though the use of animal models has given some edge, the existence of species-specific cellular interactions and the difficulty of transferring their clinical relevance to humans have undermined their legitimacy in forecasting precise outcomes in clinical trials. Technological progress in science and medicine has given rise to many in vitro BBB models to investigate different neurological ailments, including malignancies, but these lack dynamic blood flow conditions. Microfluidic systems and organ-on-chip technology have been able to recapitulate the 3D architect of complex tissues and organs, thereby offering new insights into complex cellular behavior and functions. There has been an expeditious advancement in the field of microfluidics and their use has been proven to be practical in numerous biomedical applications. The efficiency of existing BBB-on-a-chip models can further be improved through the use of hydrogels that mimic the 3D micro architecture of tissues. Hydrogel-based BBB-on-a-chip models can recapitulate a 3D microenvironment conducive for cell growth and allow for the exchange of gases and nutrients and the removal of metabolic wastes between cells and the cell/ECM interface, and thus are able to regulating ECM behavior. In addition, they enable the simultaneous assessment of drug penetration across the BBB, the efficacy of therapeutic molecules on tumor cells, and the evaluation of the toxicity profile of drugs for a normal cell in the case of an intact BBB and within the tumor-invaded cells in the case of a disintegrated BBB. Here, we have provided a comprehensive review of currently available BBB-on-chip models, focusing on the device design type, incubated cell types, and salient features. These models have proven or been proposed to have direct involvement in brain primary and secondary malignancies. We further discussed the design concept of available hydrogel- (natural/synthetic) based in vitro microfluidic BBB models.

However, different cell types respond differently when grown over a microfluidic chip. Various complex device designs and a plethora of cell types make it very difficult to generalize any concept. Further, suboptimal cell growth in different chambers of the microfluidic chip can also be an issue that needs to be taken care of when making an overall assessment of the obtained outcomes. As most of the generated cell-specific information is optimized, analyzed, and evaluated on glass or polystyrene based culture plates, most microfluidic chips are fabricated using PDMS, and a straightforward comparison on the cellular behavior on these discrete substrates is very difficult. This increases the time and utilization of resources involved in regenerating and evaluating findings. Another challenge that needs to be adequately handled is the maintenance of sterility conditions for the designed organ-on-a-chip. This ensures the consistency and reliability of the obtained results. Furthermore, as microfluidic devices handle a very minute volume of samples, absolute care is required during the execution and handling process.

Organ-on-a-chip technology demands a highly interdisciplinary approach and a collective effort from biologists, biochemists, chemists, material sciences, and engineers. To see through the production of microfluidic devices from laboratory table to clinical platforms, their cost effectiveness in terms of mass production, their reliability, and the reproducibility of the generated data are all crucial aspects. Effort needs to be made to pursue and fabricate novel materials that can be used to provide long term culture support. The subsequent application of hydrogels as the materials of choice and the recognition of in vitro hydrogel-based BBB-on-chip models by regulatory authorities and industries could lead to the enhanced throughput screening of chemotherapeutic agents and assist in further gaining insight into brain malignancies. This will also aid in the diminution of the use of animal models frequently used in BBB-related research.

## Figures and Tables

**Table 1:** Summary of the existing BBB-on-chip models for oncology research.

S. No	Device Designs	Fabrication Method	Cell Type	Features	Ref.
1.	Polydimethylsiloxane (PDMS) parallel	Soft lithography	Human iPS cell-derived endothelial cells (iPSC-ECs)/human PCs/human ACs	Highly functional 3D BBB in vitro model; produced by vasculogenesis	[102]
2.	PDMS parallel	Soft lithography	Human brain-derived microvascular endothelial cells (TY10 cell line)	High resolution 3D live fluorescence and TEM imaging; miniature design to fit for advanced lattice light sheet microscopy	[103]
3.	PDMS parallel	Soft lithography	Rat brain endothelial cell line (RBE4)	Upregulation of tight junction molecules; formation of intact functional BBB model	[104]
4.	PDMS sandwich (X-shaped microchannels)	Spin coating	Mouse b.End3 ECs cells and C8-D1A astrocytes	TEER levels typically exceeded 250 Ω cm^2^ in co-culture BBB model; change in TEER values in response to histamine exposure was observed in real-time	[95]
5.	ECM gel-based tubular structure	Soft lithography	Rat brain ECs (RBE4)	Localization at endothelial cell boundaries of ZO-1 and VE-Cadherin; adequate for detailed functional studies of BBBs; applicable for screening BBB-targeting drugs	[105]
6.	Polyethylene terephthalate (PET) parallel	3D lithography	Human vascular ECs/bovine brain PCs /immortalized human umbilical vein ECs (HUVECtert2)	Enabled 3D localization microscopy of the cytoskeleton; 3D single-molecule-sensitive tracing of lipoprotein particles	[106]
7.	PDMS parallel	Soft lithography	Human brain microvascular ECs (hBMVECs)/human ACs	Lumens generated with no delamination; level of secretary proteins much higher than the Transwell model; use of the designed model to identify the contribution of individual cell types	[98]
8.	PDMS sandwich (S-shaped microchannels)	Soft lithography	bEnd.3 cells (mouse)	Analysis on the effect of flow on targeting and penetration of nanoparticles; investigation on the transcellular versus paracellular transport tested at different fluid shear stress	[94]
9.	PDMS parallel	Soft lithography	HUVECs/human ACs	Measurement of permeability values; investigation of the effect of hydrogen peroxide on the trans-endothelial permeability	[107]
10.	Glass slide parallel	Soft lithography	Human brain microvascular ECs (HBMVEC)	Identified protein kinase C-delta (PKCδ) as a critical regulatory of inflammatory response; PKCδ can alter the physiology and functioning of the BBB	[108]
11.	PDMS parallel (S-shaped microchannels)	Stereolithography	iPSCs-derived brain microvascular endothelial-like cells (BMECs)/ACs/neurons	Precisely predicted blood-to-brain permeability of pharmacologics; capable of advanced drug screening and personalized medicine	[77]
12.	Organoplate parallel	Photolithography	Human induced pluripotent stem cells (hiPSCs) BMECs/rat primary ACs	Highest TEER levels reported so far (above 4000 Ω cm^2^ on day 3 and sustained above 2000 Ω cm^2^ up to 10 days); applicable for efficient drug screening; capable of simulating multi-organ interactions on drug response	[100]
13.	PDMS parallel	Photolithography	Human ACs (HA-1800)/(hBMVEC)	Multi-organ microfluidic chip to study brain metastasis; reported the elevation of the expression of Aldo-keto reductase family 1 B10 (AKR1B10) in lung cancer brain metastasis	[109]
14.	GelMA	3D Bioprinting	Mouse macrophages cell line (RAW264.7)/mouse glioblastoma cells (GL261)	Analysis on the connection between glioblastoma-associated macrophages (GAM) and glioblastoma cells (GC); in vitro model to mimic the interaction of GAM and GC; the applicability of the model for evaluating novel cancer therapeutics	[110]
15.	Decellularized ECM from patient brain	3D Bioprinting	Patient-derived tumor cells, vascular ECs	Mimicking the biochemical, structural, and biophysical properties of the native tumors; applicability of the model to determine drug combinations for enhanced tumor killing	[111]

**Table 2:** Overview of hydrogel-based BBB models for brain malignancy.

S. No	Type of Brain Malignancy	Chemotherapeutic Agents/Cells	Targeting Pathway	Type of Hydrogel	Ref.
1.	Glioblastoma	Human umbilical vein endothelial cells (HUVECs)	RTK/Ras pathway	Collagen 1	[142]
2.	Glioblastoma	U-251 MG cells		Collagen I	[143]
3.	Brain metastasis	Temozolomide (TMZ)/BMECs, ACs		Collagen gels	[144]
4.	Glioblastoma and brain metastasis	Mouse brain endothelial cells (bEnd.3)		Collagen	[145]
5.	Glioblastoma and brain metastasis	Primary human brain microvascular ECs, primary brain ACs, PCs		Collagen	[78]
6.	Glioblastoma multiforme (GBM)	Pitavastatin and irinotecan/glioblastoma cells (U87)		Poly(ethylene) glycol diacrylate (PEGDA)	[147]

## Data Availability

No new data were created or analyzed in this study.
